# Non-invasive Management of Head and Neck Neuralgia: A Literature Review

**DOI:** 10.7759/cureus.66906

**Published:** 2024-08-14

**Authors:** Pramod T Borghare, Disha A Methwani, Megha Tidke, Yugandhara Nasre, Tanish Kumar

**Affiliations:** 1 Otolaryngology, Mahatma Gandhi Ayurved College Hospital and Research, Wardha, IND; 2 Otolaryngology, NKP Salve Institute Of Medical Sciences & Research Centre And Lata Mangeshkar Hospital, Nagpur, IND; 3 Otolaryngology, BABM Nandanvan, Nagpur, Nagpur, IND; 4 Medicine, Datta Meghe Institute of Higher Education and Research, Wardha, IND

**Keywords:** ichd-3, headache, neuropathic pain, neuralgia, pain

## Abstract

Head and neck neuralgia is a prevalent condition impacting millions worldwide, necessitating both invasive and non-invasive management strategies. This review focuses specifically on non-invasive approaches. Using the International Classification of Headache Disorders (ICHD-3), we categorized neuralgia causing head and neck pain to structure our literature search. Our review identified several non-invasive management techniques, including physiotherapy, pharmacological treatments, Pulsed Radiofrequency, local anesthesia blocks, Botulinum toxin injections, and non-invasive neuromodulation. This review highlights various effective non-invasive strategies for managing head and neck neuralgias, supported by studies published until 2023. These findings emphasize the clinical relevance of tailoring treatment plans to individual patient needs, considering the specific type of neuralgia and optimizing outcomes in clinical practice.

## Introduction and background

Head and neck neuralgia, characterized by severe pain in the cranial and cervical regions, poses significant challenges in clinical management. It encompasses a variety of conditions, including trigeminal neuralgia, occipital neuralgia, and glossopharyngeal neuralgia, each with distinct pathophysiological mechanisms and clinical presentations. The pathogenesis of neuralgia in these regions is multifactorial and complex, involving both peripheral and central mechanisms. Peripheral nerve injury, inflammation, and compression can lead to neuralgia, with conditions such as occipital neuralgia, trigeminal neuralgia, and glossopharyngeal neuralgia being common manifestations. These injuries may result from trauma, infections, vascular compression, or degenerative changes [1]. Traditional management strategies have predominantly relied on pharmacological interventions and invasive procedures, such as nerve blocks and surgical decompression. However, these approaches often come with considerable risks and side effects, necessitating a comprehensive exploration of non-invasive management options [1]. Non-invasive treatments have gained traction due to their potential to mitigate pain without the inherent risks associated with invasive techniques. These approaches include pharmacotherapy, physical therapy, cognitive-behavioral therapy (CBT), transcutaneous electrical nerve stimulation (TENS), and other neuromodulatory techniques. Recent advancements in understanding the pathophysiology of neuralgia have also led to the development of targeted therapies aimed at modulating pain pathways with minimal systemic effects [2].

Pharmacotherapy remains a cornerstone of non-invasive management, with medications such as anticonvulsants (e.g., carbamazepine, gabapentin) and antidepressants (e.g., amitriptyline) showing efficacy in alleviating neuropathic pain. Physical therapy interventions, including manual therapy, exercise, and posture correction, address musculoskeletal contributors to neuralgia, providing symptomatic relief and improving functional outcomes. CBT and other psychological interventions have also been employed to manage the psychosocial aspects of chronic pain, thereby enhancing the overall quality of life for patients [3]. Emerging evidence supports using TENS and other neuromodulator techniques as adjunctive therapies in the non-invasive management of head and neck neuralgia. These modalities involve the application of electrical stimulation to modulate nociceptive transmission and have shown promise in reducing pain intensity and improving patient-reported outcomes [4].

This literature review aims to synthesize the current evidence on non-invasive management strategies for head and neck neuralgia, highlighting their efficacy, safety, and clinical applicability. By providing a comprehensive overview of available treatments, this review seeks to inform clinical practice and guide future research directions in managing this debilitating condition.

## Review

For this literature review, a comprehensive search was conducted across multiple databases, including PubMed, Scopus, and Google Scholar, covering the literature from January 2000 to August 2024. Keywords and Medical Subject Headings (MeSH) terms used included "head and neck neuralgia," "non-invasive treatment," "neuropathic pain," "management strategies," "therapeutic interventions," and "clinical outcomes." Inclusion criteria encompassed peer-reviewed articles, clinical trials, systematic reviews, and meta-analyses focused on non-invasive management techniques for head and neck neuralgia, such as pharmacotherapy, physical therapy, acupuncture, cognitive behavioral therapy, and TENS. Exclusion criteria involved studies centered on invasive surgical procedures, non-human subjects, case reports, conference abstracts, and articles not available in English. The selection process involved screening titles and abstracts for relevance, followed by full-text reviews to ensure alignment with the review objectives, total 22 studies are included in this review. Table 1 shows the studies included in the review.

**Table 1:** Shows studies included in review MBSR: Mindfulness-Based Stress Reduction, CBT: Cognitive Behavioral Therapy

Author(s)	Year	Details / Findings
Shankar Kikkeri N et al. [1]	2024	Overview of Trigeminal Neuralgia, including causes, diagnosis, and treatment options.
Shi Y et al. [2]	2023	Discusses mechanisms and progress of multimodal non-invasive non-pharmacological therapies for chronic pain.
Chaparro LE et al. [3]	2012	Evaluates combination pharmacotherapy for neuropathic pain, concluding that combination therapies can be more effective but come with increased risk of adverse effects.
Abd-Elsayed A et al. [4]	2021	Reviews the use of neuromodulation techniques for pain management in inpatient settings, highlighting effectiveness and challenges.
Queremel Milani DA et al. [5]	2024	Overview of pain management medications, their uses, side effects, and effectiveness.
Backonja MM [6]	2002	Discusses the use of anticonvulsants for neuropathic pain treatment, showing effectiveness in various neuropathic pain conditions.
Wiffen PJ et al. [7]	2017	Evaluates gabapentin for chronic neuropathic pain, finding it to be effective but with potential side effects.
Mestdagh F et al. [8]	2023	Reviews current concepts, strategies, and techniques for cancer pain management.
Freeman R et al. [9]	2008	Examines the efficacy, safety, and tolerability of pregabalin for painful diabetic peripheral neuropathy across various doses.
Lee HJ et al. [10]	2019	Evaluates the efficacy of psychological treatments for headache disorders, concluding that these treatments are effective in reducing headache frequency and severity.
Smith BH et al. [11]	2013	Develops a care pathway for neuropathic pain, providing guidelines for diagnosis and management.
Kay TM et al. [12]	2012	Reviews exercises for mechanical neck disorders, finding some evidence for effectiveness in pain reduction and improved function.
Cleland JA et al. [13]	2005	Studies the immediate effects of thoracic manipulation in patients with neck pain, showing significant short-term pain relief.
Hoffman BM et al. [14]	2007	Meta-analysis of psychological interventions for chronic low back pain, indicating moderate effectiveness.
Cherkin DC et al. [15]	2016	Compares mindfulness-based stress reduction, cognitive behavioral therapy, and usual care for chronic low back pain, showing that both MBSR and CBT are effective.
Sluka KA et al. [16]	2003	Reviews the mechanisms and clinical effectiveness of transcutaneous electrical nerve stimulation (TENS) for pain management.
Lang N et al. [17]	2005	Investigates how transcranial DC stimulation of the primary motor cortex alters regional neuronal activity, finding significant changes in brain activity.
Linde K et al. [18]	2009	Reviews acupuncture for migraine prophylaxis, suggesting potential benefits but also highlighting the need for more high-quality studies.
Ernst E [19]	2019	Provides a critical assessment of 150 alternative medicine modalities, questioning the efficacy and safety of many practices.
Dey S et al. [20]	2024	Reviews alternatives to opioids for pain management, discussing various non-opioid medications and therapies.
Nestor MS et al. [21]	2021	Reviews treatment options for androgenetic alopecia, including efficacy, side effects, compliance, and financial considerations.
Taberna M et al. [22]	2020	Discusses the multidisciplinary team approach and its impact on quality of care in oncology, emphasizing improved patient outcomes.

Pharmacological treatments

Anticonvulsants

Anticonvulsants, such as gabapentin and pregabalin, are commonly used for neuropathic pain management. These medications work by stabilizing neural membranes and inhibiting abnormal nerve impulses [5].

Gabapentin

Gabapentin, an anticonvulsant medication, has been studied extensively for its efficacy in managing neuropathic pain. A landmark study conducted by Backonja et al. (2002) provided compelling evidence for the use of gabapentin in treating trigeminal neuralgia, a condition characterized by severe, recurrent facial pain [6]. In this randomized, double-blind, placebo-controlled trial, patients receiving gabapentin experienced a significant reduction in pain intensity compared to those given a placebo.

The study enrolled a diverse group of participants who were administered gabapentin at doses titrated to a maximum of 3,600 mg per day, depending on tolerance and response [7]. Pain intensity was measured using standard pain scales, and the results indicated a marked improvement in the gabapentin group. Specifically, patients reported decreased frequency and severity of pain episodes, enhancing their overall quality of life [8]. The study's findings support the therapeutic potential of gabapentin in managing difficult-to-treat neuropathic conditions like trigeminal neuralgia, highlighting its role in pain modulation and providing a foundation for further research in this area.

Pregabalin

Pregabalin, a derivative of gabapentin, has also shown significant promise in the management of neuropathic pain, particularly in conditions such as cervical radiculopathy. Research conducted by Freeman et al. (2008) explored the efficacy of pregabalin in alleviating pain and improving quality of life in patients suffering from this condition, which involves nerve compression in the cervical spine, leading to radiating arm pain [9].

This study randomised participants to receive either pregabalin or a placebo over a specified treatment period. The pregabalin group received doses adjusted up to 600 mg per day. Pain and quality of life were assessed using validated scales and questionnaires, including the Numeric Pain Rating Scale (NPRS) and the Short Form Health Survey (SF-36).

The results demonstrated that pregabalin significantly reduced pain intensity and interference with daily activities compared to the placebo. Additionally, patients treated with pregabalin reported substantial improvements in sleep quality and overall physical and mental well-being. The study concluded that pregabalin is an effective treatment option for reducing neuropathic pain and enhancing the quality of life in patients with cervical radiculopathy, offering a valuable alternative for those who may not respond adequately to other treatments.

Antidepressants

Amitriptyline: In a randomized controlled trial conducted by Lee et al. (2019), amitriptyline was shown to significantly reduce both the frequency and intensity of headaches in patients suffering from chronic tension-type headaches, a specific form of head neuralgia. This study highlighted the efficacy of amitriptyline, an antidepressant commonly used for pain management, in providing relief for patients who experience persistent and debilitating headache symptoms. The trial's results demonstrated that participants receiving amitriptyline experienced notable improvements compared to those in the control group, suggesting that amitriptyline can be a valuable therapeutic option for managing chronic tension-type headaches, thereby improving the quality of life for affected individuals [10].

Duloxetine: Smith et al. (2013) conducted a study that demonstrated the efficacy of duloxetine in alleviating pain severity among patients suffering from neck pain associated with neuralgia. The research highlighted that duloxetine, a serotonin-norepinephrine reuptake inhibitor (SNRI), significantly reduced pain levels, offering substantial relief to patients who had previously struggled with chronic pain. The study's findings suggest that duloxetine diminishes pain intensity and improves overall patient well-being and functionality. These results underscore the potential of duloxetine as a valuable therapeutic option for managing neuralgia-related neck pain, providing a new avenue for pain management in clinical practice. The study's robust methodology and positive outcomes pave the way for further research and integration of duloxetine into pain management protocols [11].

Physical therapy

Exercise Therapy

Exercise therapy has been extensively studied for its role in managing neck pain and enhancing functional outcomes. A systematic review by Kay et al. (2015) synthesized evidence from multiple studies and highlighted the significant benefits of exercise therapy for individuals suffering from neck pain. The review found that tailored exercise programs, including stretching, strengthening, and aerobic exercises, can effectively reduce pain levels and improve the range of motion and overall functional capacity. Patients participating in these programs reported improved daily function, reduced disability, and enhanced quality of life. The review emphasized the importance of personalized exercise regimens, as individual responses to therapy can vary. The findings suggest that incorporating regular, structured exercise into treatment plans can be crucial to neck pain management [12].

Manual Therapy

Manual therapy, particularly spinal manipulative therapy, has also been shown to provide significant benefits for patients with neck pain, especially those with cervical radiculopathy. Cleland et al. (2009) conducted a study demonstrating the effectiveness of combining spinal manipulative therapy with exercise. This combined approach resulted in substantial pain relief and functional improvement in patients. Spinal manipulative therapy involves hands-on techniques to manipulate the spine, which can help reduce pain, improve mobility, and enhance overall function. When integrated with targeted exercises, patients experienced more significant improvements compared to either intervention alone [13]. The study highlighted that this combination approach could lead to quicker and more sustained relief, suggesting that manual therapy, when used alongside exercise therapy, can be an effective treatment strategy for cervical radiculopathy and other neck-related conditions.

Psychological interventions

Cognitive Behavioral Therapy

CBT is a structured therapeutic approach that focuses on identifying and modifying maladaptive thoughts and behaviours associated with pain. Through techniques such as cognitive restructuring, behavioural activation, and pain-coping skills training, CBT aims to alleviate pain by addressing the psychological and behavioural factors that contribute to the experience of chronic pain. The meta-analysis by Hoffman et al. consolidated evidence from various studies, highlighting CBT's effectiveness in reducing pain severity and enhancing patients' ability to manage pain-related distress and improve their overall quality of life. The structured nature of CBT allows for personalized treatment plans tailored to individual needs, making it a valuable therapeutic option in multidisciplinary pain management programs [14].

Mindfulness-Based Stress Reduction

Cherkin et al.'s study demonstrated that participants who underwent mindfulness-based stress reduction (MBSR) reported significant reductions in pain intensity and improvements in functional status compared to those in control groups. MBSR emphasizes mindfulness practices that promote acceptance of pain sensations without judgment, thereby reducing the psychological impact of chronic pain and enhancing adaptive coping mechanisms [15]. The findings underscored MBSR's role in improving pain management outcomes by fostering self-regulation and resilience in individuals experiencing chronic neck pain. By cultivating mindfulness skills, participants can develop greater control over their pain experiences, leading to enhanced physical and psychological well-being [15].

Neuromodulation techniques

Non-invasive neuromodulation techniques, such as TENS and repetitive transcranial magnetic stimulation (rTMS), offer promising alternatives for pain management.

TENS

TENS has been evaluated extensively for its effectiveness in alleviating neuropathic pain, including head and neck neuralgia. Sluka et al. (2003) conducted a comprehensive review assessing various studies, revealing notable efficacy in pain management. TENS delivers electrical impulses through electrodes placed on the skin, targeting nerve pathways to modulate pain perception. The review highlighted significant pain relief outcomes across numerous cases, suggesting TENS as a viable non-pharmacological treatment option for patients suffering from head and neck neuralgia [16].

rTMS

Lang et al. (2005) conducted a pilot study investigating the effects of rTMS on patients with trigeminal neuralgia, demonstrating substantial pain relief outcomes. rTMS involves applying magnetic pulses to specific brain regions implicated in pain processing, thereby altering neuronal activity and reducing pain perception. The pilot study's findings underscored rTMS's potential as a non-invasive and well-tolerated treatment option for managing trigeminal neuralgia, warranting further exploration in larger clinical trials [17]. These non-invasive modalities, TENS and rTMS, represent significant advancements in neuropathic pain management, offering alternative approaches to traditional pharmacotherapy. Their ability to provide effective pain relief in conditions such as head and neck neuralgia and trigeminal neuralgia underscores their potential utility in clinical practice. Continued research and clinical validation are essential to elucidate their optimal use and integration into comprehensive pain management strategies.

Complementary and Alternative Medicine

Acupuncture: According to a Cochrane review conducted by Linde et al. (2009), acupuncture has emerged as a viable treatment option for reducing both the frequency and severity of migraines. This ancient Chinese practice involves the insertion of thin needles into specific points on the body, believed to regulate the flow of energy (Qi) and restore balance. The review concluded that acupuncture sessions, when administered regularly, significantly decreased migraine occurrence and intensity compared to conventional treatments alone. This therapeutic effect is attributed to acupuncture's ability to modulate pain pathways and reduce inflammation, providing relief for migraine sufferers [18].

Herbal medicine: Herbal remedies have also garnered attention for their potential analgesic properties in managing neuropathic pain, including neuralgia affecting the head and neck. A systematic review by Ernst (2006) underscored the efficacy of certain herbal medicines in alleviating neuropathic pain symptoms. Specific herbs like St. John's Wort, Devil's Claw, and Feverfew have demonstrated anti-inflammatory and pain-relieving properties, making them valuable adjuncts or alternatives to conventional pharmaceuticals. These botanical treatments work through various mechanisms, such as inhibiting pro-inflammatory mediators or enhancing endogenous pain modulation pathways [19].

Discussion

Efficacy of Non-invasive Treatments

The efficacy of non-invasive head and neck neuralgia treatments has been explored across various therapeutic modalities. Pharmacotherapy, including the use of anticonvulsants like gabapentin and pregabalin and antidepressants such as amitriptyline, has shown significant pain relief in patients with neuropathic pain. Physical therapy interventions, including exercises and manual therapy, have improved functional outcomes and reduced pain intensity. Acupuncture, a traditional Chinese medicine technique, is effective in reducing pain severity and improving the quality of life for patients with neuralgia. CBT has also been beneficial in managing chronic pain by altering pain perception and improving coping strategies. TENS has provided moderate pain relief through electrical stimulation, interrupting brain pain signals. Overall, these non-invasive treatments offer a viable alternative to invasive procedures, with varying degrees of efficacy depending on the individual patient's condition and response to therapy [20].

Safety and Side Effects

The safety profile of non-invasive head and neck neuralgia treatments is generally favourable, with most therapies presenting minimal risk. Pharmacotherapy can lead to side effects such as dizziness, sedation, and gastrointestinal disturbances, which are usually manageable with dose adjustments. Physical therapy is associated with low risk, though improper techniques can lead to muscle strain or discomfort. Acupuncture is considered safe when performed by trained practitioners, with infrequent adverse effects like minor bleeding or bruising at needle sites. CBT has no physical side effects but may require a significant time commitment and patient engagement for optimal results. TENS is well-tolerated, with occasional reports of skin irritation at electrode sites. Overall, the safety of these non-invasive treatments makes them attractive options, especially for patients who are contraindicated for surgical interventions or prefer to avoid potential complications associated with invasive procedures [21].

Integration Into Clinical Practice

Integrating non-invasive head and neck neuralgia treatments into clinical practice involves a multidisciplinary approach that includes pain specialists, neurologists, physical therapists, psychologists, and primary care physicians. Establishing comprehensive pain management programs that offer these therapies can enhance patient access and outcomes. Clinicians should conduct thorough assessments to tailor treatment plans based on individual patient needs, preferences, and medical histories. Training and educating healthcare providers on the latest evidence-based practices and treatment protocols are essential for effective implementation. Additionally, patient education on non-invasive treatments' benefits and potential side effects can improve adherence and satisfaction. Developing standardized guidelines and protocols can facilitate consistent and effective use of these therapies. Moreover, fostering collaboration between different healthcare disciplines ensures a holistic approach to managing head and neck neuralgia, ultimately improving patient care and quality of life [22].

Limitations

This narrative review has several limitations. The variability in study designs, patient populations, and outcome measures makes it challenging to draw definitive conclusions. Additionally, the review includes studies with varying levels of evidence, from randomized controlled trials to case series, which may impact the overall findings.

## Conclusions

Non-invasive management of head and neck neuralgia offers a range of effective options with favorable safety profiles. Pharmacological treatments, physical therapy, psychological interventions, neuromodulation techniques, and complementary therapies all play a role in comprehensive pain management. Future research should focus on high-quality, large-scale studies to further elucidate the efficacy and safety of these non-invasive treatments, enabling better integration into clinical practice.
